# Progress in the Puzzle Resolution: The Molecular Genetics Underpinning Autism Spectrum Disorders

**DOI:** 10.3390/brainsci13121688

**Published:** 2023-12-07

**Authors:** Alessandra Mezzelani, Francesca Anna Cupaioli

**Affiliations:** National Research Council, Institute for Biomedical Technologies, Via Fratelli Cervi 93, 20054 Segrate, Italy; alessandra.mezzelani@itb.cnr.it

Autism spectrum disorders (ASDs) are neurodevelopmental conditions characterized by impairments in social interaction, communication, and the presence of restricted, repetitive behaviors. The etiology of ASDs is complex, with both genetic and environmental factors playing key roles in the onset of these conditions. However, the molecular genetics of ASDs has drawn the attention of the scientific community due to its profound implications in understanding the molecular processes underlying the disorders and their implications for diagnosis, patient stratification, intervention, and ultimately, amelioration of life quality for individuals with an ASD. In fact, 65–90% of ASDs phenotypic variance can be explained by genetic risk factors [1].

Indeed, in recent decades, research into the molecular basis of ASDs has significantly broadened our understanding of how genetic factors contribute to the development and variability of autistic traits. This Special Issue aimed to offer a comprehensive overview of the current state of knowledge regarding the molecular genetics linked to ASDs, illustrating how this understanding is crucial for advancing towards more personalized and effective interventions for individuals with these disorders.

A significant advancement in understanding the genetic architecture of ASDs has been the identification of numerous risk-conferring genes. The SFARI database has reported 71 high-confidence genes (https://gene.sfari.org, (accessed on 4 October 2023)) and more than one thousand of risk genes. High-throughput sequencing technologies, including whole-exome and whole-genome sequencing, have played a pivotal role in the discovery of several genes linked to ASDs. Furthermore, copy number variants (CNVs) and single nucleotide polymorphisms (SNPs) have also been identified as significant molecular mechanisms contributing to the genetics of ASDs.

Moreover, ASDs exhibit a substantial level of genetic heterogeneity. There are common variants conferring a small degree of risk, along with rare mutations which can significantly increase susceptibility to the condition. This vast genetic heterogeneity translates into diverse phenotypes within the autism spectrum, thus explaining the ‘spectrum’ nature of autism [2].

Research has also explored the epigenetic modifications and their role in ASDs. Epigenetic alterations, such as DNA methylation and histone modifications, can modulate gene expression without altering the genome, providing another layer of complexity to the genetic basis of this condition [3]. Recent studies have shed light on a variety of epigenetic modifications associated with ASDs, further emphasizing the intricate interplay between genetic and epigenetic mechanisms in this disorder.

Investigation into the molecular pathways disrupted in ASDs has also been at the forefront of research. These pathways, including those involved in synaptic transmission, neuronal signaling, and immune responses, contribute to the broader neurobiological understanding of ASDs. Uncovering how genetic mutations and epigenetic modifications impact these pathways is crucial for the development of targeted therapeutic strategies.

The integration of genetic findings with other omics technologies, such as transcriptomics, proteomics, metagenomics, and metabolomics, is paving the way towards a more holistic understanding of ASDs. The developing of ASD animal and 3D in vitro models are also helping in disentangle molecular mechanisms implicated in the condition. By elucidating the downstream effects of genetic alterations on RNA transcriptions, protein profiles, and metabolic levels, researchers are beginning to put the pieces of the ASD puzzle together.

While remarkable progress has been made, the molecular genetic landscape of ASDs has still not been fully elucidated. There is a need for larger collaborative efforts and cross-disciplinary research to further unravel the complex genetic architecture of ASDs. The integration of genetic, epigenetic, and omics technologies is leading the scientific community towards a more refined comprehension of ASDs, and more efficacious and personalized interventions in the near future.
